# Association of Time to Definitive Hemostasis With Mortality in Patients With Solid Organ Injuries

**DOI:** 10.7759/cureus.45401

**Published:** 2023-09-17

**Authors:** Michaela Wycoff, Thomas P Hoag, Raymond I Okeke, John T Culhane

**Affiliations:** 1 General Surgery, MercyOne Des Moines Medical Center, Des Moines, USA; 2 General Surgery, Saint Louis University School of Medicine, Saint Louis, USA

**Keywords:** selection bias in trauma, survival bias, golden hour of trauma, mortality and hemostasis, angioembolization, solid organ injury, surgical hemostasis, time to hemostasis, golden hour

## Abstract

Introduction

The Golden Hour is a term used in the trauma setting to refer to the first 60 minutes after injury. Traditionally, definitive care within this period was believed to dramatically increase a patient’s survival. Though the period of 60 minutes is unlikely to represent a point of distinct inflection in survival, the effect of time to definitive care on survival remains incompletely understood. This study aims to measure the association of time to definitive hemostasis with mortality in patients with solid organ injuries as well as the effect of survival bias and a form of selection bias known as indication by severity on the relationship between time to treatment and survival.

Methodology

This is a retrospective cohort study using data obtained from the American College of Surgeons National Trauma Data Bank (NTDB) from the years 2017 through 2019 selecting patients treated for blunt liver, spleen, or kidney injury who required angioembolization or surgical hemostasis within six hours. A Cox proportional hazards regression was used to analyze time to death. The association of probability of death with time was examined with a multivariate logistic regression initially treating the relationship as linear and subsequently transforming time to hemostasis with restricted cubic splines to model a non-linear association with the outcome. To model survival and indication by severity bias, we created a computer-generated data set and used LOESS regressions to display curves of the simulated data.

Results

The multivariate Cox proportional hazards analysis shows a coefficient of negative 0.004 for minutes to hemostasis with an adjusted hazard ratio of 0.9959 showing the adjusted hazard of death slightly diminishes with each increasing minute to hemostasis. The likelihood ratio chi-square difference between the model with time to hemostasis included as a linear term versus the model with the restricted cubic spline transformation is 97.46 (p<0.0001) showing the model with restricted cubic splines is a better fit for the data. The computer-generated data simulating treatment of solid organ injury with no programmed bias displays an almost linear association of mortality with increased treatment delay. When indications by severity bias and survival bias are introduced, the risk of death decreases with time to hemostasis as in the real-world data.

Conclusion

Decreasing mortality with increasing delay to hemostasis in trauma patients with solid organ injury is likely due to confounding due to indication by severity and survival bias. After taking these biases into account, the association of delayed hemostasis with better survival is not likely due to the benefit of delay but rather the delay sorts patients by severity of injury with those more likely to die being treated first. These biases are extremely difficult to eliminate which limits the ability to measure the true effect of delay with retrospective data. The findings may however be of value as a predictive model to anticipate the acuity of a patient after an interval of unavoidable delay such as with a long transfer time.

## Introduction

Blunt abdominal trauma is a major cause of morbidity and mortality in the United States. The most commonly injured solid organs are the liver and spleen, followed by the kidney and pancreas [1]. Solid organ injuries are successfully managed by observation in 83-95% of cases; however, some patients require procedural intervention. Surgery is indicated for hemodynamic instability and an independent need for exploratory laparotomy [1]. Relative indications include a higher grade of injury and the quantity of hemoperitoneum. Procedural interventions include exploratory laparotomy and angioembolization [2,3].

Intuitively one would expect prompt interventions to be more beneficial. This is a familiar concept in trauma known colloquially as the Golden Hour, referring to the first 60 minutes after injury in which achieving homeostasis has been thought to dramatically increase survival. 

Recent evidence has exposed the golden period of precisely 60 minutes as largely a myth but the genuine effect of time to definitive care on survival remains incompletely understood. Some studies have shown a direct correlation between delayed time to hemostasis and increased morbidity while others have aroused skepticism showing minimal correlation between survival and treatment delay [4].

This study has two aims: first to measure the association of time to definitive hemostasis with mortality in patients with solid organ injuries. Measuring the probability of survival continuously over time will allow us to identify any inflection of the survival curve at one hour or any other time. Second, we will examine the effect of survival bias and a form of selection bias known as indication by severity bias on the relationship between time to treatment and survival. Our hypothesis is that shorter time to hemostasis is associated with improved survival in blunt solid organ injury patients who require procedural hemostasis.

## Materials and methods

Study design

The first part of the analysis is a retrospective cohort study measuring the association of mortality with time to hemostasis for solid organ injuries. The next part of the analysis is a computer simulation created to analyze the shape of the survival curve.

Data collection

Data for this analysis were obtained from the American College of Surgeons National Trauma Data Bank (NTDB), which compiles patient information from over 900 trauma centers across the United States. The years 2017 through 2019 were chosen.

Inclusion and exclusion criteria

We selected patients with blunt liver, spleen, or kidney injury who required angioembolization or surgical hemostasis within six hours. We selected the six-hour time frame because early trauma deaths are generally due to bleeding; hence, the mortality for these patients should be mostly influenced by the timing of hemostasis. Limiting the analysis to the early period also makes the data less skewed and eliminates outliers. We use the time of arrival at the hospital as time zero. The time of trauma is not available in the NTDB, and transport times are recorded less consistently than hospital arrival. 

Statistical analysis

Descriptive data are displayed for baseline characteristics and the distribution of time to hemostasis. To analyze time to death, we used a Cox proportional hazards regression with the outcome of mortality. Time to hemostasis was entered as a time-dependent variable. Fixed covariates include age, sex, injury severity score (ISS), initial systolic blood pressure, liver, spleen, and kidney severity scores, and units of packed red blood cells and plasma transfused within the first four hours.

Examination of the non-linear association of the probability of death with time to hemostasis was performed with multivariate logistic regression. We used a simplified model with death as the outcome and time to hemostasis as a predictor of interest, controlling for covariates of sex, age, and ISS. Covariates were chosen based on a priori importance and on the Wald test for the contribution of individual variables to the model.

The logistic regression was performed initially treating the probability of death versus time to hemostasis as linear with no transformation. Restricted cubic splines with five knots were then used to transform the predictor of interest (time to hemostasis) to a model involving a non-linear association with the outcome. We used this regression to generate a line graph with confidence intervals displaying the relationship between time to hemostasis and the probability of survival. To compare the goodness of fit of the models with time to hemostasis as linear versus non-linear terms, we calculated the likelihood ratio chi-square difference between the model with time to hemostasis included as a linear term versus the model with the restricted cubic spline transformation. Statistics were performed with R and reviewed with the department statistician.

Computer simulation

Potential biases arise when the severity of the injury influences the time to hemostasis. To model these biases, we next created a computer-generated dataset. A Python script was used to simulate a cohort of 12000 patients of whom 9000 are actively bleeding. Time of observation is divided into 36 intervals. We specify a mortality per interval between 1 and 5 percent with random variation. All patients underwent a hemostasis procedure. The time to procedure is set to random times across the range of intervals. The procedure reduces the mortality per interval by 95%. All patients are included in the dataset even if they die before the procedure. 

We then used the computer-simulated data to demonstrate the effects of survival bias and of confounding by indication by severity of injury. First, we introduced the indication by severity bias. We simulated this by dividing the time to hemostasis by the percent mortality per interval. Thus, the time to hemostasis is proportionately shorter for injuries with a greater risk of death. This simulates providers acting faster for more severe injuries and selecting sicker patients for more prompt treatment.

Next, survival bias was introduced by eliminating all the patients who died before scheduled hemostasis. Patients with more lethal injuries die sooner, thus those still alive and present at later intervals tend to have less serious injuries. Finally, we combined the simulated indication by severity and survival biases into a single analysis.

To display simulated data, we use LOESS regressions to smooth the curves of the generated plots. We do not require a mathematical model such as logistic regression with splines for this purpose because we are not interested in making inferences about the simulated data but only in showing the visual changes in the curves.

## Results

Nine thousand sixteen patients with a solid organ injury required a hemostatic procedure (HP). Tables 1, 2 show the baseline characteristics of this population. 

**Table 1:** Baseline characteristics of selected patients including sex, category of solid organ injury, and treatment modality.

Characteristic	n	Percent
Death	2539	28.2
Male sex	6023	66.8
Liver injury	4564	50.6
Spleen injury	6144	68.1
Kidney injury	2154	23.9
Angioembolization	2487	27.6
Laparotomy	6529	72.4

**Table 2:** Baseline characteristics of selected patients including age, blood pressure, time to hemostatic procedure, injury severity score adjusted to organ category, and blood products.

Characteristic	Mean	Standard Deviation
Age	42.2	19.3
Initial systolic blood pressure	107.4	30.2
Minutes to angiography	188.2	155.1
Minutes to laparotomy	92.1	61.5
Injury severity score	33.7	14.0
Abbreviated injury severity score		
Liver	1.5	1.7
Spleen	2.3	1.8
Kidney	0.7	1.3
Blood products over the first 4 hours		
Packed red cells	8.2	9.1
Plasma	5.6	7.6
Platelets	1.4	3.6

**Figure 1:**
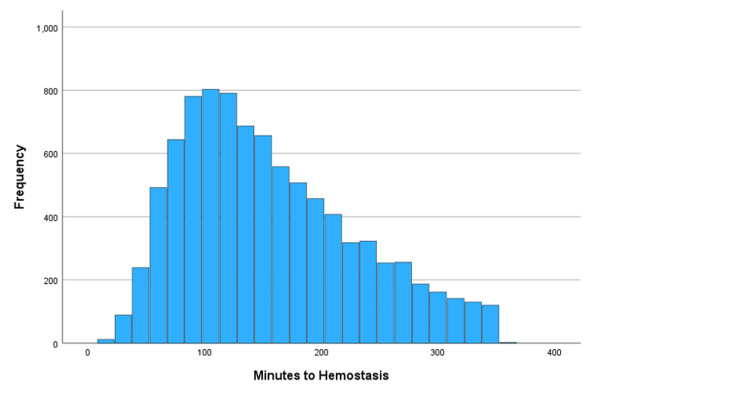
Time to the HP ranging from 15 to 350 minutes with the most number of patients receiving treatment at 100 minutes. Frequency represents the number of patients. HP: Hemostatic procedure

Figure 1 shows the distribution of the time until the HP.

The multivariate Cox proportional hazard analysis with admission to hemostasis as a time-dependent variable with the additional covariates listed in the methods shows a coefficient of negative 0.004 for minutes to hemostasis. The adjusted hazard ratio is 0.9959. 95% confidence interval is (0.9952 to 0.9966, p<0.001). The likelihood ratio test is 1947 on 10 degrees of freedom (p=<0.001). The coefficient represents the expected change in log hazard of death associated with a one-minute increase in time to HP. The adjusted hazard ratio is the adjusted hazard of death with each increasing minute of time to HP. The negative coefficient shows that the adjusted hazard of death slightly diminishes with each increasing minute to HP.

The multivariate logistic regression that treats the probability of death versus time to hemostasis as linear with no transformation shows a coefficient of negative 0.0054 for time to hemostasis. Standard error of the coefficient is 0.0004. The odds ratio of death for each increasing minute to HP is 0.995. Comparing the odds of death at each interquartile boundary shows the adjusted odds of death at 211 minutes versus 104.4 minutes is 0.56 (p<0.001 for all values). 

The model that uses restricted cubic spline transformation of time to hemostasis to account for non-linear probability of outcome over time shows a coefficient of negative 0.0185 for minutes to hemostasis. Standard error of the coefficient is 0.0025. The odds ratio of death for each increasing minute to HP is 0.982. Comparing the odds of death at each interquartile boundary shows that the adjusted odds of death at 211 minutes versus 104.4 minutes is 0.53 (p<0.001 for all values). As in the Cox analysis, the odds of death diminishes with time to HP for the multivariate logistic regressions.

The likelihood ratio chi-square difference between the model with time to hemostasis included as a linear term and the model with the restricted cubic spline transformation is 1367.53-1270.07 = 97.46. This chi value corresponds to a p-value of <0.0001. This shows that the model with restricted cubic splines is a better fit for the data.

**Figure 2:**
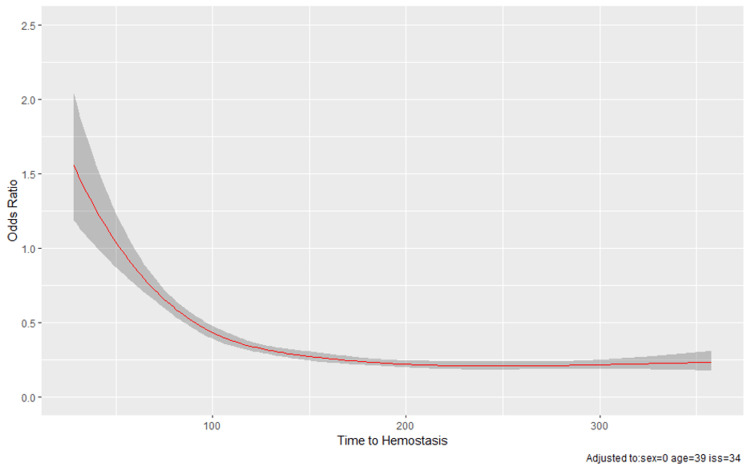
Predicted odds ratio of death versus time to hemostasis adjusted to sex, age, and ISS. Multivariate model including restricted cubic spline transformation of the time to hemostasis. ISS: Injury severity score

Figure 2 shows the predicted odds of death versus time to hemostasis using the model of the non-linear effect of time to hemostasis.

**Figure 3:**
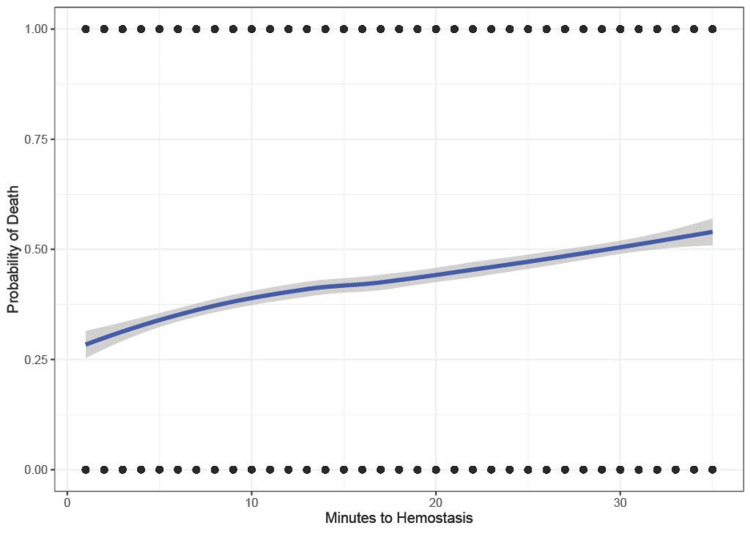
Computer-generated data simulating treatment of solid organ injury with no simulated bias. Probability of death ranging from 0 to 1 versus time to hemostasis ranging from 0 to 35 minutes. We use minutes for simplicity, but the units could be any uniform time interval.

Figure 3 shows computer-generated data simulating treatment of solid organ injury. This initial simulated course of treatment includes no programmed survival or indication by severity bias. The probability of death increases with time to hemostasis. The curve is almost linear with a little deviation due to random variation. 

**Figure 4:**
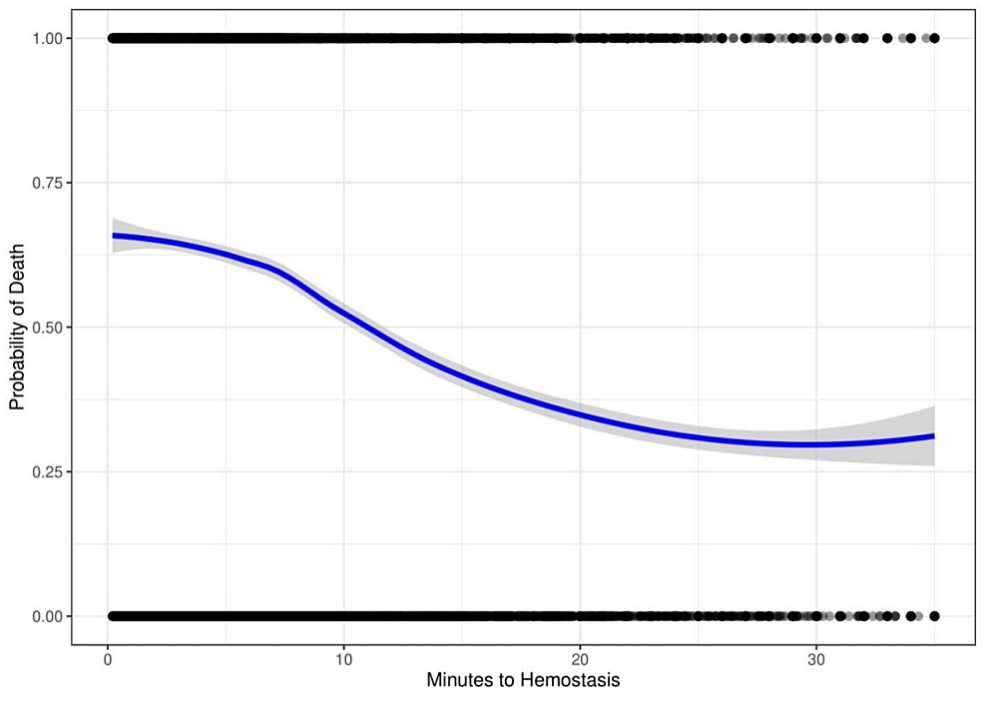
Computer-generated data showing the risk of death versus time to hemostasis with simulated indication by severity bias. The probability of death is 65% at 0 minutes to hemostasis and decreases to 30% at 35 minutes to hemostasis.

Figure 4 shows the result of indication by severity bias introduced into the simulated data. Time to hemostasis is adjusted to be shorter for injuries with a higher probability of death. In this simulation, the risk of death decreases with time to hemostasis.

**Figure 5:**
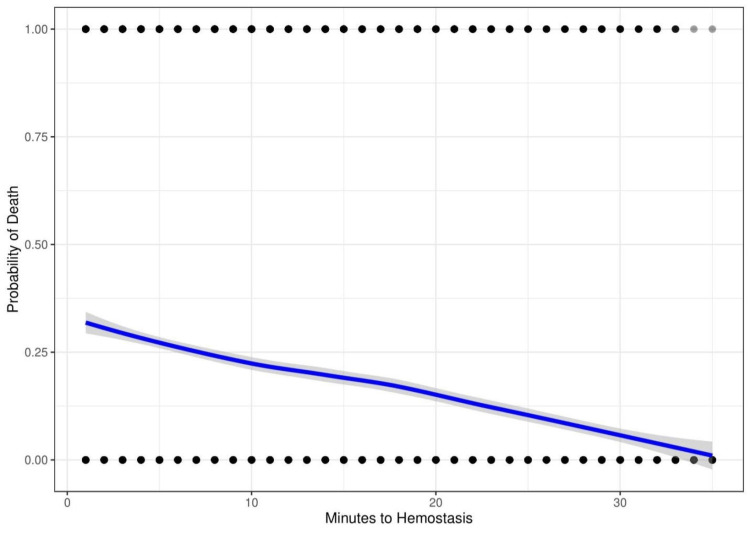
Computer-generated data showing the risk of death versus time to hemostasis with simulated survival bias. The probability of death is 36% at 0 minutes to hemostasis and decreases to 0% at 35 minutes to hemostasis.

Figure 5 displays a simulated survival bias. Patients who died before they could obtain the HP were eliminated. This leaves a less seriously injured cohort alive to get later treatment. Survival bias also converts the slope of the probability of death versus time to hemostasis to negative, showing a decreased risk of death with time to hemostasis.

**Figure 6:**
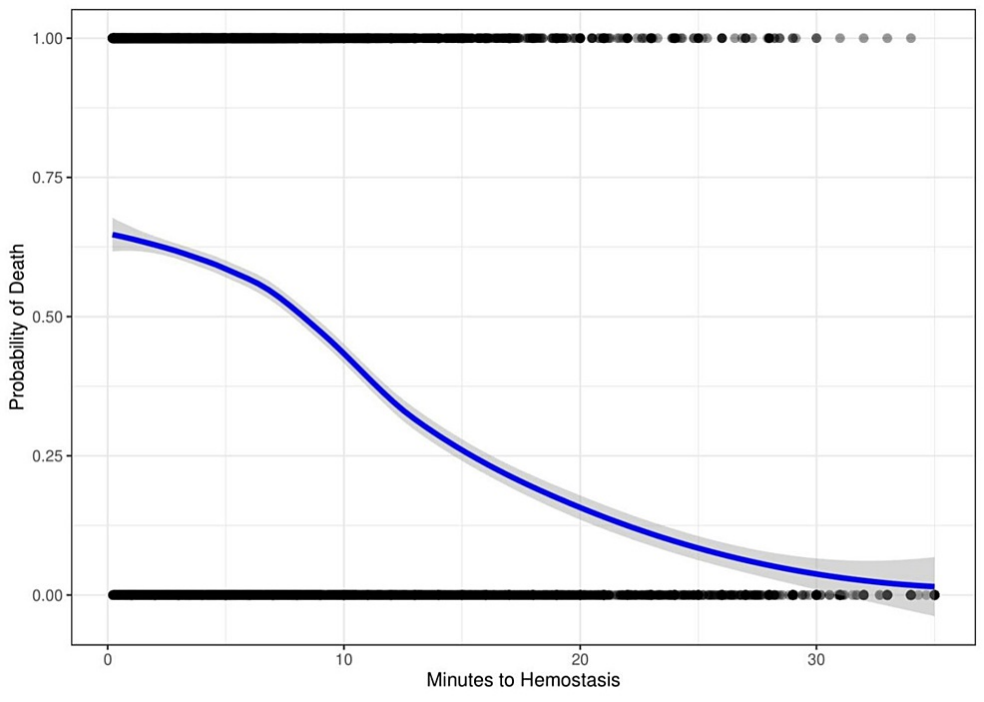
Computer-generated data showing the risk of death versus time to hemostasis with both simulated indication by severity and survival bias. The probability of death is 63% at 0 minutes to hemostasis and decreases to ~0% at 35 minutes to hemostasis.

Figure 6 shows the computer-generated data when indication by severity and survival bias are applied together. There is a negative relationship between time to hemostasis and death.

## Discussion

The term Golden Hour was coined in the 1970s by military physician Robert A. Cowley after his experience treating injured soldiers in a field trauma setting. Dr. Cowley used his experience in combat to pilot numerous trauma centers around the United States, ultimately founding the Shock Trauma Institute in Baltimore. It was his expertise in prioritizing transport and triage that helped give rise to the trauma care system we know today. His “Golden Hour” concept was originally described as a golden period which referred to the first six hours after injury; however, now it is commonly thought of as the first 60 minutes after injury. Cowley argued that achieving hemodynamic stability within the golden period gave trauma patients a dramatically higher chance of survival [5]. Dr. Cowley advanced the golden period idea in support of his plan to regionalize trauma centers to decrease delay to definitive treatment. His idea persisted and has been cited as dogma for years [6].

While it is logical that prompt treatment should benefit trauma patients, the literature shows an inconsistent relationship between time to definitive treatment and survival. Some studies show that shorter delay is associated with better survival [4,7-11]. Others show no association of survival with time to treatment [12-15]. Still, others show that longer delay was associated with better survival for undifferentiated trauma. For instance, in a systematic review of prehospital on-scene time, Harmsen et al. showed an association of improved survival with increased delay [16]. He attributed this to the value of prehospital interventions. In an NTDB study from 2019, Chen et al reported findings similar to ours for undifferentiated trauma. They found that mortality was increased for patients whose prehospital time was 30 minutes or less [17]. 

Our findings show a pattern that is almost the inverse of a golden period. The odds of death decrease with increased delay to hemostasis with an infection at about two hours. These results appear counterintuitive. A causal inference would be that delaying hemostasis improves survival, which is not medically plausible. One explanation for such heterogeneity and paradoxical results in the literature is that trauma is heterogeneous. Disparate results are expected because survival depends on many factors including the type of injury, severity of injury, duration of elapsed time, effectiveness of definitive therapy, and effectiveness of field and emergency department stabilization. In addition to complex predictive factors, biases play a role in the interpretation of survival data.

The second part of our study focuses on two biases that strongly influence outcomes in trauma: survival bias and confounding by indication by severity. Survival bias occurs when patients who are less severely injured are more likely to survive to receive treatment at a later time. This idea is explained by Anderson et al. who describe “immortal time bias” whereby all patients within a cohort who are selected for an exposure are essentially immortal until they receive that exposure. In this case, the immortal time period is the time between the trauma and the hemostatic intervention [18]. Patients who undergo the HP later experience a greater duration of this immortal time period and thus exhibit better survival.

Confounding by indication occurs when a covariate predicts both treatment and outcome [19]. When the degree of illness is the covariate, the phenomenon is known as confounding by severity. The sicker patients are selected for the more intensive treatment [20]. In the case of trauma, providers select patients who are more severely injured for faster treatment. Thus, the severity of injury predicts both the latency of treatment and mortality. This is a natural aspect of the triage system in any emergency medical setting.

These biases are known to affect the results of epidemiologic trauma research [21]. To further explore their effect on the outcome, we conducted a computer simulation to determine whether these biases can account for the shape of the non-linear inverse association. In our simulation, plotting mortality versus delay to treatment of a time-dependent injury with no adjustment shows the intuitively expected increase in mortality with time (Figure 3). After introducing simulated survival and indication by severity biases into the model, we see a curve resembling the real-world data (Figure 6). This demonstrates how biases can not only mask any potential benefit but produce the observed paradoxical negative association. 

With awareness of these biases, we can use statistical methods to reduce their effect. Stratifying comparisons by injury and employing multivariate analysis can be used to control confounding by severity. Previous studies have shown that some highly lethal injuries are time-sensitive when analyzed this way [7,9,17]. Limiting the time period of analysis may be used to reduce the magnitude of survival bias [17], as can proportional hazard analysis with time-dependent covariates. 

Our analysis as well as the analyses seen in other studies shows that we can control for some elements of selection and survival bias, however we cannot completely eliminate them. In an epidemiologic study designed to test methods of controlling indication bias, Bosco et al. analyzed the effect of adjuvant therapy on breast cancer. In describing what they termed a “most stubborn bias”, they employed multiple statistical methods to control confounding by indication but found that they could not completely eradicate imbalance between treatment groups [22].

Recognition that these biases are universal and incompletely controlled will help us interpret the measured association between time to treatment and survival. One implication is relevant to field stabilization versus rapid transport. Better survival after longer delay could be attributed to the benefit of more time devoted to field stabilization, but this conclusion may not be justified. If no treatment whatsoever were rendered in the field, the above-described biases would still produce an association between increased transport time and lower in-hospital mortality. Our results can be used as a predictive rather than explanatory model. Patients received after long prehospital time or transfer are likely to be more stable because the sicker patients have already been eliminated. This expectation could factor into triage and activation criteria. 

Limitations 

We used time of arrival rather than time of trauma as the beginning of the time to hemostasis interval. Time of arrival is a less accurate measure of total time to hemostasis, but it was more consistently recorded, and it is relevant for delays within the hospital. We did not stratify patients by the type of solid organ injury or method of hemostasis. Delays might have had a different effect on a subset of our population. The protective association of delay to HP with death is probably still influenced by selection bias with unknown/unmeasured risk factors. Time to hemostasis may be based on radiologic findings, hemodynamic trends, initial lab values, response to volume resuscitation, pressor requirements, and clinical indices of perfusion. There may be some residual survival bias. When the delay to procedure is not random, Cox analysis cannot control for timing of treatment due to underlying risk; thus, survival bias can still exist [23].

## Conclusions

Mortality decreased with increasing delay to the HP in trauma patients with solid organ injury. The association persisted after controlling for multiple confounders including time-dependent covariates. This counterintuitive result is likely due to confounding by severity and survival bias. Our computer simulation shows how severity and survival bias can produce a survival curve that shows a beneficial association of survival with increasing time to treatment, even when delay actually diminishes survival. Our interpretation after taking these biases into account is that delay to hemostasis does not improve survival but rather sorts the patients by severity of injury with those more likely to die being treated first.

These biases are difficult to eradicate, even with multivariate statistical models controlling for time-dependent covariates and multiple injury characteristics. More sophisticated models will help reduce these biases but not eliminate them completely. Our results may be of value as a predictive model to anticipate the acuity of a patient after a given interval of unavoidable delay such as a long transfer time. We recommend caution in the interpretation of delay to treatment as an explanation of outcomes in studies based on retrospective data.
